# A corepressor participates in LexA-independent regulation of error-prone polymerases in *Acinetobacter*

**DOI:** 10.1099/mic.0.000866

**Published:** 2019-11-05

**Authors:** Megan A. Peterson, Alison N. Grice, Janelle M. Hare

**Affiliations:** ^1^​ Department of Biology and Chemistry, Morehead State University, Morehead, KY 40351, USA; ^2^​ Office of Information Technology, University of Colorado Denver, Anschutz Medical Campus, Aurora, CO 80045, USA

**Keywords:** DNA damage, DdrR, UmuDAb, SOS response, LexA, repressor

## Abstract

The DNA damage response of the multidrug-resistant pathogen *Acinetobacter baumannii *, which induces mutagenic UmuD′_2_C error-prone polymerases, differs from that of many bacteria. *Acinetobacter* species lack a LexA repressor, but induce gene transcription after DNA damage. One regulator, UmuDAb, binds to and represses the promoters of the multiple *A. baumannii* ATCC 17978 *umuDC* alleles and the divergently transcribed *umuDAb* and *ddrR* genes. *ddrR* is unique to the genus *Acinetobacter* and of unknown function. 5' RACE (rapid amplification of cDNA ends) PCR mapping of the *umuDAb* and *ddrR* transcriptional start sites revealed that their −35 promoter elements overlapped the UmuDAb binding site, suggesting that UmuDAb simultaneously repressed expression of both genes by blocking polymerase access. This coordinated control of *ddrR* and *umuDAb* suggested that *ddrR* might also regulate DNA damage-inducible gene transcription. RNA-sequencing experiments in 17 978 *ddrR* ^−^ cells showed that *ddrR* regulated approximately 25 % (*n*=39) of the mitomycin C-induced regulon, with *umuDAb* coregulating 17 of these *ddrR*-regulated genes. Eight genes (the *umuDC* polymerases, *umuDAb* and *ddrR*) were de-repressed in the absence of DNA damage, and nine genes were uninduced in the presence of DNA damage, in both *ddrR* and *umuDAb* mutant strains. These data suggest *ddrR* has multiple roles, both as a co-repressor and as a positive regulator of DNA damage-inducible gene transcription. Additionally, 57 genes were induced by mitomycin C in the *ddrR* mutant but not in wild-type cells. This regulon contained multiple genes for DNA replication, recombination and repair, transcriptional regulators, RND efflux, and transport. This study uncovered another regulator of the atypical DNA damage response of this genus, to help describe how this pathogen acquires drug resistance through its expression of the error-prone polymerases under DdrR and UmuDAb control.

## Introduction

In many bacteria, DNA damage caused by UV light, mitomycin C (MMC) or antibiotics can induce a multitude of DNA damage response (SOS) genes that are under LexA control [1, 2]. LexA typically represses genes by binding to promoters of these genes [3] until it undergoes auto-cleavage by an activated RecA protein after DNA damage [4, 5]. Despite lacking a *lexA* gene, the *Acinetobacter baumannii* characteristics associated with DNA damage-induced mutagenesis are consistent with an SOS response: (i) repression of transcription in the absence of DNA damage [1–3], (ii) induction of RecA-facilitated repressor self-cleavage after DNA damage [6] caused by (iii) antibiotics (e.g. ciprofloxacin and tetracycline [7, 8]), UV light or MMC [9, 10], (iv) leading to the induction of antibiotic resistance (v) [7, 11, 12] in a process requiring protein synthesis.

One regulator of a minority of the DNA damage-induced genes in *Acinetobacter* species is UmuDAb. This homologue of the error-prone polymerase manager UmuD is encoded throughout the bacterial genus *Acinetobacter* [9], in addition to these species’ *umuD* genes encoded in operons with the *umuC* error-prone polymerase. UmuDAb shares with its UmuD homologues a catalytic C-terminal serine protease domain that enables its self-cleavage. Unlike *umuD*, however, the *Acinetobacter umuDAb* encodes an additional N-terminal domain that binds DNA [13] and is required for its repressor action [14]. These UmuDAb self-cleavage abilities [6], size [15] and function [14] resemble features of LexA. UmuDAb represses gene transcription until DNA damage (via antibiotics, radiation or chemical exposure [16]) triggers the induction of a subset of the typical SOS genes [17]. In *A. baumannii* ATCC strain 17 978 (hereafter abbreviated 17978), the UmuDAb-repressed regulon defined by both RNA-sequencing (RNA-seq) and microarray studies is notable for including the six error-prone Y-family polymerase *umuDC* genes (A1S_2008, 2015, 0636–637, 1173–1174) that contribute to SOS mutagenesis [10, 17]. The other members of the UmuDAb regulon include *umuDAb* itself (A1S_1389), and the *ddrR* gene (A1S_1388) transcribed divergently from it [13, 17].

We previously identified *ddrR* as a UmuDAb-regulated, DNA damage-inducible gene in *Acinetobacter baylyi* [15]. Its 246 bp ORF codes for 81 amino acids in *A. baylyi* ADP1 (78 amino acids in 17978 cells), and is preceded by an appropriately spaced ribosome binding site in both species. It is present in virtually all *Acinetobacter* species, including the pathogens *A. baumannii* and *A. ursingii* [9]. When present, it is always transcribed divergently from *umuDAb*; *umuDAb* is similarly always next to *ddrR. ddrR* shares a putative, ~282 bp promoter region with *umuDAb* in *A. baumannii* strains. The inclusion of *ddrR*, which is not encoded by microbes outside the genus *Acinetobacter *, in the UmuDAb regulon, as well as the conserved genomic organization of the *umuDAb–ddrR* gene pair within *Acinetobacter* species, suggested that it might play a role in the UmuDAb-mediated, atypical SOS response of this genus.

**Fig. 1.**
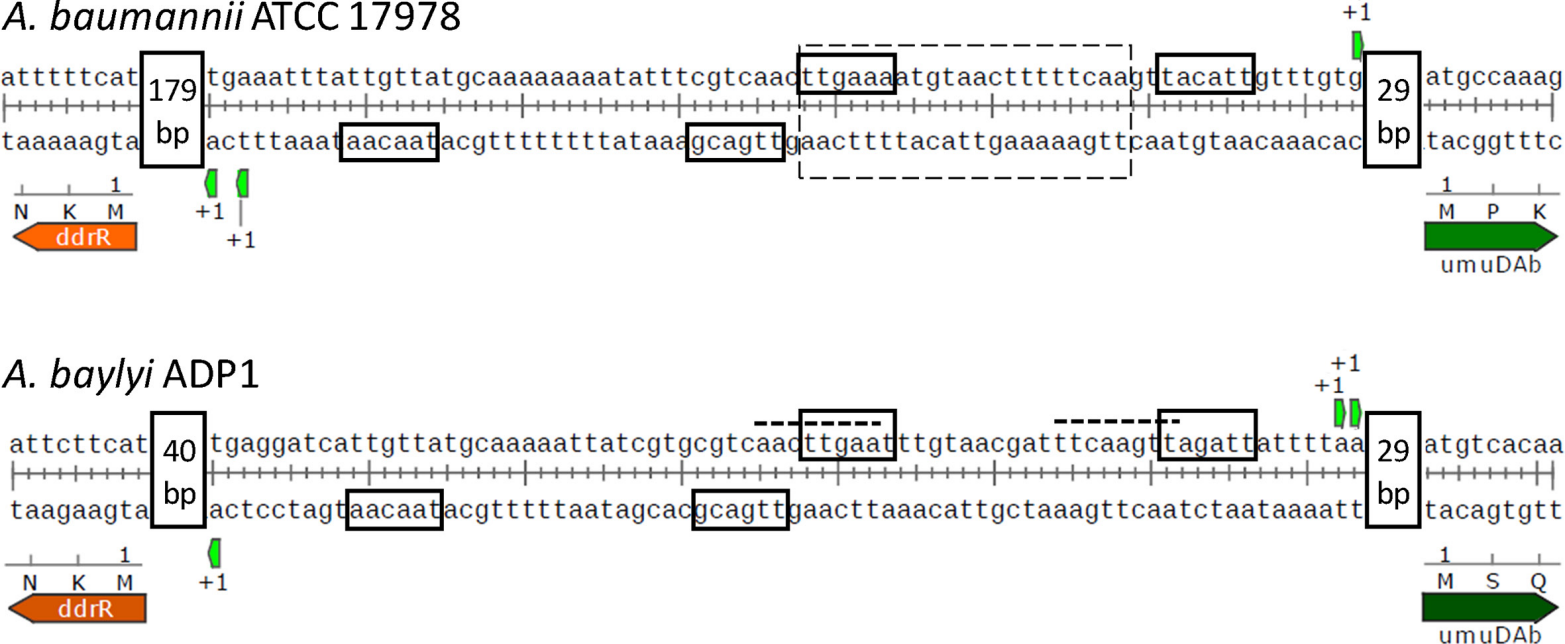
5' RACE (rapid amplification of cDNA ends) analyses reveal the relationship of the UmuDAb binding region to the *ddrR-umuDAb* promoters. 5' RACE experiments were conducted on RNA from MMC-treated 17978 and ADP1 cells. Upon mapping the first (+1) mRNA base of each gene, the −35 elements of each gene were observed to overlap either the defined UmuDAb binding site (for 17978) or an inverted repeat that, when mutated, abolished transcription of both genes in ADP1 cells [14]. The dotted box marks a UmuDAb binding site defined in 17978 through gel shift experiments [13], the dashed lines indicate the inverted repeats proposed to be regulatory binding sites in ADP1 [18], and the solid boxes represent −10 and −35 promoter consensus elements suggested by the +1 transcriptional start site(s). Distances between the +1 transcriptional start sites and the coding regions are shown in vertical boxes.

We hypothesized that inverted repeats in the *umuDAb–ddrR* intergenic region in *A. baylyi* (Fig. 1) served as a binding site for a regulatory protein [18]. Aranda *et al*. later identified a nearly identical palindromic sequence between *umuDAb* and *ddrR* in 17978 cells and demonstrated that UmuDAb binds to this DNA sequence in their promoters as well as to putative promoter regions upstream of the other *umuDC* homologues in 17 978 [13]. Mutations in the shared *umuDAb–ddrR* promoter region of *A. baylyi* significantly alter the transcription of both genes in a coordinated fashion [14]. Mutations in the most proximal (relative to *umuDAb*) region of the palindromic sequence abolish the repression typical of *ddrR* and *umuDAb* transcription under non-inducing conditions, and result in constitutive, high expression of both genes. However, mutations in the region most distal to *umuDAb* prevent the induction of transcription even under DNA-damaging conditions.

One need to decipher DNA damage regulatory mechanisms in *Acinetobacter* is that the opportunistic pathogen *A. baumannii* creates DNA damage-induced resistance to antibiotics that are used clinically [10]. As this species has been identified as a ‘serious threat’ to human health by the Centers for Disease Control [19] and other agencies given its multi-drug and pan-drug resistance, it is necessary to understand how its DNA damage response contributes to this problem. Furthermore, understanding how this genus induces ~150 genes after DNA damage [10, 13, 17], without encoding a *lexA* repressor gene [9], will advance our knowledge of novel transcriptional regulatory mechanisms.

We investigated the mechanism by which both *ddrR* and *umuDAb* were coordinately regulated by a promoter region that contained only one UmuDAb binding site. We further hypothesized that *ddrR*, like UmuDAb, was required for regulation of DNA damage-inducible genes. RNA-seq experiments in *A. baumannii* 17978 cells tested whether *ddrR* regulates any DNA damage-inducible genes, only UmuDAb-regulated genes or all of them. We found that in this species, all UmuDAb-repressed genes were corepressed by DdrR, and other UmuDAb-regulated genes required DdrR for their full induction after DNA damage. Finally, we saw that DdrR might participate in additional regulatory networks, as it regulated both DNA damage-inducible genes that do not require UmuDAb action and genes that were only induced in *ddrR* mutant cells.

## Methods

### Bacterial strains and growth conditions

All *Acinetobacter* strains described in Table 1 (* A. baylyi* strains derived from ADP1 and *A. baumannii* strains derived from ATCC 17978) were grown at 37 °C in minimal media plus succinate [18] for RNA-seq transcriptome and real-time quantitative PCR (RT-qPCR) analyses. As described previously [17], for both RNA-seq transcriptome and RT-qPCR analyses, a 3 ml overnight culture, grown at 37 °C at 250 r.p.m., was diluted 1 : 25 into 5 ml of fresh medium and grown with shaking for 2 h, at which time the culture was split in two and 2 μg MMC ml^−1^ was added to one culture. This concentration of MMC was chosen to correspond to that used in previous studies, including RNA-seq studies, to facilitate direct comparison between results. [When measuring *benA* induction, sodium benzoate (3 mM) was added to the medium [18], instead of MMC.] Further incubation for 3 h allowed gene expression before isolation of total RNA. Kanamycin was added to LB medium at 30 μg ml^−1^, and gentamycin was added at 20 μg ml^−1^ for selection after transformation.

**Table 1.** Strains used in this study

Strain name	Genotype	Characteristics	Reference
* Acinetobacter baylyi* strain ADP1	Wild type		
* Acinetobacter baumannii* ATCC 17978	Wild type		ATCC [24]
ACIAD2730	ADP1 −53_−1del*ddrR* Δ*ddrR*::*tdk/kan*	Km^r^	[21]
ACIAD0535	ADP1 Δ*clpX*::*tdk*/*kan*	Km^r^; source of the *tdk*–KanR cassette	[21]
JH1700	17978 *ddrR*:Tn*LK*	Km^r^	This study
* A. baylyi* MSUcds2730	ADP1 Δ*ddrR*::*tdk/kan*	Km^r^	This study
* A. baylyi* DR-Stop	ADP1 *ddrR* 10C>T	DdrR Q4X	This study

### Construction of a 17978 *ddrR::lacZkanR* mutant

An *A. baumannii* ATCC 17978 *ddrR::lacZ* insertion mutant was constructed by allelic replacement of the wild type (WT) allele in a series of steps. First, a custom *lacZ-kanR* transposon was constructed and used in an *in vitro* transposition reaction, where it randomly transposed into *ddrR* contained in the plasmid p17UDDR. p17UDDR was constructed by PCR-amplifying a 2.8 kbp fragment of 17978 chromosomal DNA with primers Out17UDAbRev and Out17UDAbFor, and cloning the PCR product into pGEM-T Easy (Promega). p17UDDR encodes A1S_1389 (UmuDAb; genomic coordinates 1 641 271–1 631 882 in GenBank CP000521.1), A1S_ 1388 (DdrR; genomic coordinates 1 630 752–1 630 988), and hypothetical proteins A1S_1390 and A1S_3662. The transposon was constructed by ligating a 4.7 kb *Pst*I fragment of pKOK6 [20], which contains a promoterless *lacZ*-kanamycin resistance gene cassette, into the *Pst*I site of the EZ::TN pMOD-3<R6Kγ*ori*/MCS>Transposon Construction Vector (Epicentre) to produce plasmid pMOD3*LK*. This plasmid contained a 5.5 kb transposon that was named Tn*LK*, and which was excised from pMOD3*LK* by *Psh*AI digestion. Equimolar amounts of Tn*LK* and the target plasmid p17UDDR were mixed with 1 U of EZ::TN transposase (Epicentre) and incubated at 37 °C for 2 h to allow *in vitro* transposition of Tn*LK* into p17UDDR.

Second, the transformation of this transposition mixture into *Escherichia coli* DH5α yielded the plasmid pTn14, which contained Tn*LK* inserted after the 46th base pair of *ddrR*, oriented with *lacZ* in the same orientation as *ddrR* (determined by DNA sequencing). Third, an 8.2 kb *Spe*I*–Nco*I fragment from pTn14 was subcloned into the *Sma*I site of the suicide vector pEX18Gm, which contains the *sacB* counter-selectable marker for recombination. Fourth, the resulting plasmid, pEXTn14, was electroporated into 17978 cells and selected for on gentamycin- and kanamycin-containing medium. Gentamycin- and kanamycin-resistant transformants were grown for 3 h in LB at 37 °C before plating on kanamycin- and 5 % (w/v) sucrose-containing LB agar for counterselection. PCR and DNA sequencing analyses of gentamycin-sensitive, kanamycin- and sucrose-resistant transformants confirmed that allelic replacement of the wild type *ddrR* allele with the insertion mutation had occurred and that the cells were not merodiploids. This strain was named JH1700.

### *A. baylyi* mutant strain construction

A Δ*ddrR* strain of ADP1 cells, MSUcds2730, was constructed in which the 246 bp *ddrR* ORF was replaced with the *tdk*/*kan* cassette that de Berardinis *et al*. previously used to make a library of single-gene deletion mutants of all genes in ADP1 [21]. In that library's *ddrR* mutant strain (ACIAD2730), 53 bp upstream of the *ddrR* ORF (that begins with a methionine codon and is preceded by a ribosome-binding site) was also deleted. This may have been a mis-annotation of the ORF, as the 53 bp are an ORF in ADP1 cells. (There is no similar ORF upstream of the *A. baumannii ddrR*.) Current annotations indicate a 246 bp *ddrR* ORF at base pairs 2 674 651–2 674 896 in the ADP1 genome GenBank file CR543861.1. To avoid possible interference with the promoter of *ddrR*, we deleted only the 246 bp *ddrR* ORF to form *A. baylyi* strain MSUcds2730.

Three PCR products were amplified from *A. baylyi* genomic DNA with NEB Long Amp Polymerase. PCR amplification with the primer pairs (i) ADP1drUDRevRC and 2730DStdk/KanFor, and (ii) CL-5 and 2730UStdk/KanRev was used to amplify the regions flanking the *ddrR* gene. The primer pair TDKKanFor and TDKKanRev used *A. baylyi* strain ACIAD0535 (Δ*clpX::tdk*/*kan*) as a source of the *tdk*–KanR cassette [21]. These three products were combined in equimolar amounts and used in splicing-overlap extension PCR amplification with primers CL-5 and ADP1drUDRevRC. The resulting 3.3 kb PCR product was transformed into ADP1 cells and selected for on LB-kanamycin plates. PCR and DNA sequencing confirmed the mutation.

The *A. baylyi* strain DR-Stop was constructed through two-piece splicing-overlap extension PCR to contain a nonsense (ochre) mutation of amino acid 4 of the ADP1 *ddrR* coding region. PCR amplification of ADP1 genomic DNA with Long Amp Polymerase and primer pairs (i) CL-4 and ADP1StopddrRFor, and (ii) To81Rev and ADP1StopddrRRev produced two products that were used in equimolar amounts in a third PCR with primer pair CL-4 and To81Rev. This 1.7 kb PCR product was transformed into *A. baylyi* strain ACIAD2729 (*umuDAb::tdk*-KanR) and plated on LB plates containing azithromycin at 200 μg ml^−1^ to counter-select for allelic replacement of the *tdk*-KanR cassette of *umuDAb* (ACIAD2729) with the mutated DNA PCR product. Azithromycin-resistant colonies were screened for kanamycin resistance, and kanamycin-sensitive colonies were tested with PCR and DNA sequencing to confirm the presence of the stop codon in *ddrR*. Strains, plasmids and primers are described in Tables 1–3, respectively.

**Table 2.** Plasmids used in this study

Plasmid	Description	Source/reference
pKOK6	Source of promoterless *lacZ*-Km^r^ gene cassette, with bidirectional transcriptional stop *t* sequence inserted between *lacZ* and Km^r^; Km^r^ Amp^r^	[20]
pEX18Gm	Counterselectable suicide vector containing *sacB*; Gm^r^	[38]
pGEM-T Easy	Cloning vector; Amp^r^	Promega
EZ::TN pMOD-3<R6Kγ*ori*/MCS>	Transposon construction vector; Amp^r^	Epicentre
p17UDDR	2.8 kbp of 17978 chromosomal DNA (1 630 006–1 632 787) cloned into TA vector pGEM-T Easy; Amp^r^	This study
pMOD3*LK*	pMOD-3<r6Kγ*ori*/MCS> vector containing *lacZ-kanR* cassette cloned into *Psh*AI site to form transposon Tn*LK*; Km^r^ Amp^r^	This study
pTn14	p17UDDR containing *ddrR* 46ins47Tn*LK*; Km^r^ Amp^r^	This study
pEXTn14	pEX18Gm containing ~8 kbp *Spe*I*–Nco*I insert from pTn14 cloned into *SmaI*; Gm^r^ Km^r^	This study

**Table 3.** Primers used in this study

Primer	Purpose	Sequence
Out17UDAbRev	Amplify 17978 chromosomal DNA	CGGTAGCGACTTATAATTTT
Out17UDAbFor	Amplify 17978 chromosomal DNA	ACTCAGTGATAGATAATCGG
ddrRRACE#2	*ddrR* 5′ RACE (ADP1)	GATTACGCCAAGCTTGCATGTAGCTCTTGGGCATAACC
ddrRRACE#3	*ddrR* 5′ RACE (ADP1)	GATTACGCCAAGCTTCGTCATAATATGCTCGGCTTGTTCGG
New5RaceumuDAb	*umuDAb* 5′ RACE (ADP1)	GATTACGCCAAGCTTCGCAATCACGATATCACCTGCTTTGGCCG
5RACEudabADP1	*umuDAb* 5′ RACE (ADP1)	GATTACGCCAAGCTTCTCGACATGCTCCTGTGCAGGTGATGG
17 978umuDAbRACE	*umuDAb* 5′ RACE (17978)	GATTACGCCAAGCTTCCACACTTTAGGGGGCTGAAATTGGG
17 978ddrRRACE	*ddrR* 5′ RACE (17978)	GATTACGCCAAGCTTGAGTGGGTAAGGGGATGTAAGCC
CL-5	Construction of MSUcds2730 strain	AGATCACGAGTTCTTGACC
2730UStdk/KanRev		TTTTTATGATTTGAATTGGAGGCTGGGTTTAAACTCCCTATCAGAAATT
2730DStdk/KanFor		CGATGAGTTTTTCTAAGCATGCGGAGCTGGATCTGGGTTTATTTTAGAGTAAC
ADP1drUDRevRC		CACAACAAATGACTGGACTT
TDKKanFor	Amplification of *tdk*-KanR cassette	CCCAGCCTCCAATTCAAAT
TDKKanRev		CCAGCTCCGCATGCTTAG
CL-4	Construction of *ddrR* nonsense mutation in DR-Stop strain	CCTGCTTATGCAATGACAG
To81Rev		CTGAACGTATTTGATTGAGC
ADP1StopddrRFor		TTGCATCACGTTAATTCTTCATT
ADP1StopddrRRev		AATGAAGAATTAACGTGATGCAA

### RT-qPCR

Total RNA was purified from biological triplicates of 3 ml samples, processed with the Epicentre MasterPure RNA Purification kit and re-suspended at 200 ng µl^−1^. Further removal of contaminating DNA was performed using the Ambion DNA-*free* ‘rigorous’ DNase treatment. RT-qPCR was performed on an ABI 7300 Real-Time PCR system, as previously described [17], using primers described previously [14, 17] and in Table S1 (available in the online version of this article). Primers for A1S_1388 were designed after the transcriptional start of *ddrR* was determined; the reverse primer therefore comprised base pairs 19–41 of the *ddrR* ORF, located before the Tn*LK* insertion between base pairs 46 and 47.

### RNA-seq analyses

The Genomics Facility at the University of Louisville prepared RNA libraries with 1–2 µg of total 17978 RNA samples, using the Illumina TruSeq Stranded Total RNA LT Sample Prep Kit-Set B and the Ribo-Zero Gram-Negative Bacteria Kit for rRNA depletion and RNA fragmentation. Library validation (final fragment size: ~260 bp) was performed qualitatively on an Agilent Bioanalyzer using the Agilent DNA 1000 Kit. Sequencing library quantification was performed by a standard curve method of qPCR using the KAPA Library Quantitation Kit for Illumina Platforms DNA standards.

The samples were sequenced as 75 bp paired-end reads on the University of Louisville Center for Genetics and Molecular Medicine’s Illumina NextSeq 500 using the Nextseq500 Mid Output Kit (150 cycles), yielding an average of 18 million aligned paired-end reads per sample. Reads were mapped to GenBank reference sequences CP000521 (the 17 978 chromosome), CP000522 (plasmid pAB1) and CP000523 (plasmid pAB2) using TopHat2 alignment approaches. Differential expression was analysed with the Tuxedo suite using Cufflinks (v. 2.1.1) [22]. The transcript abundance (FPKM; fragments per kilobase of transcript per million fragments mapped reads) was calculated, and genes were considered differentially expressed after MMC treatment using CuffDiff analyses with a false discovery rate (FDR) cutoff value of *q*<0.01, and if they were induced (or repressed) more than two-fold. Sequence datasets were submitted to the NCBI Gene Expression Omnibus under accession number GSE104741. Locus tags with the prefix ‘A1S_’ refer to *A. baumannii* ATCC 17978 genes; the prefix ‘ACIAD’ indicates genes of *A. baylyi* strain ADP1.

### 5′ RACE

5′ RACE PCR was used to determine +1 sites for *umuDAb* and *ddrR* transcripts. mRNA was purified from MMC-treated ADP1 total RNA samples with Epicentre’s Terminator 5′-Phosphate-Dependent Exonuclease. Poly-A+ RNA from MMC-treated 17 978 total RNA samples was prepared with Takara PolyA polymerase before use in 5′ RACE reactions. These mRNA-enriched or poly-adenylated samples were used in the Clonetech SMARTer 5′/3′ RACE kit protocol to generate RACE-ready cDNA and perform 5′ RACE. Touchdown PCR cycling parameters based on primer melting temperatures were used for both species samples. Primers used to perform 5′ RACE reactions are listed in Table 3. All RACE PCR products were gel-purified and cloned into the Clontech linearized pRACE vector and sequenced (three clones for *umuDAb* in ADP1 and four plasmid clones for all other reactions).

### Statistical analyses

**Fig. 2.**
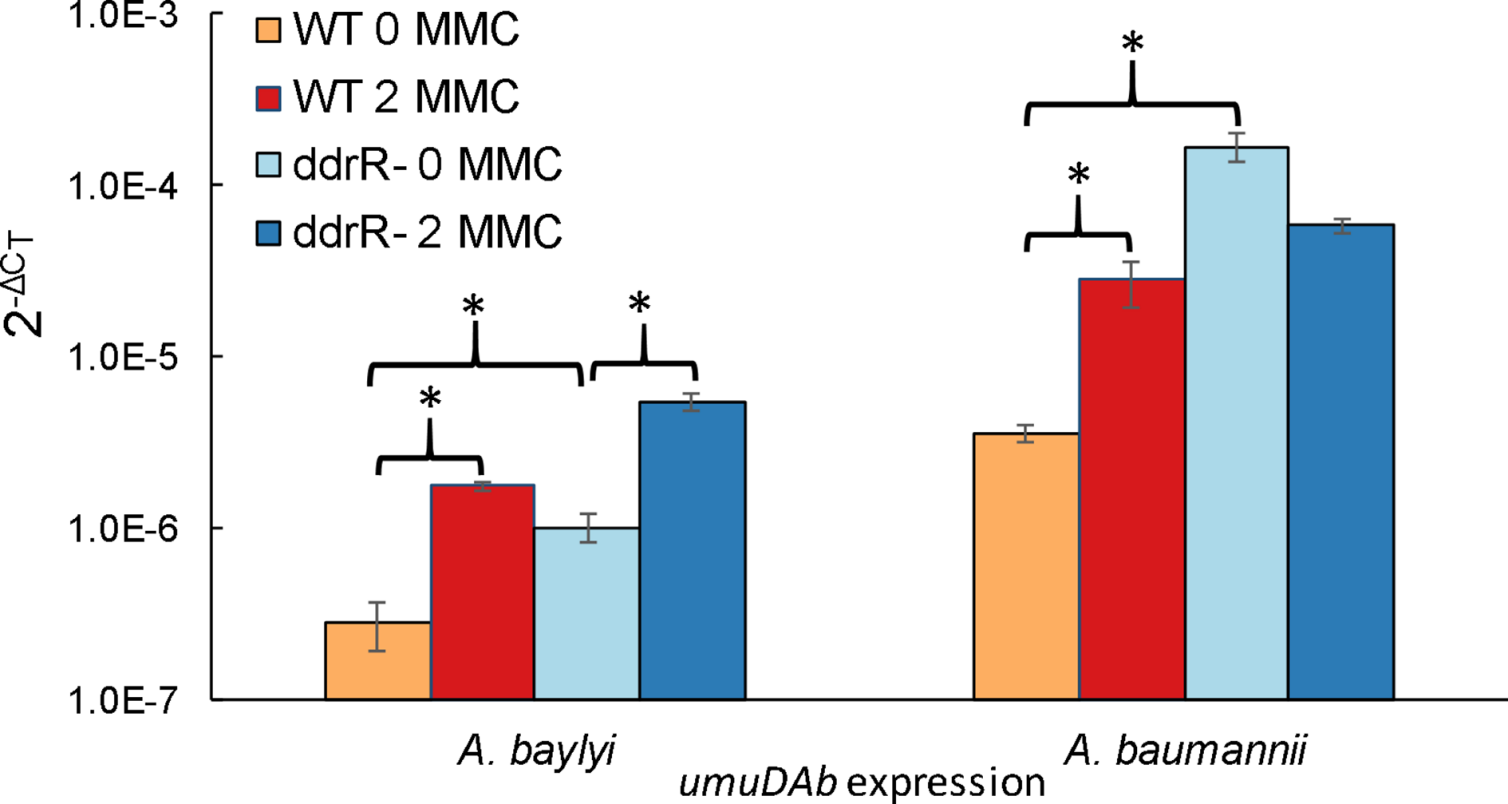
*ddrR* regulates expression of the UmuDAb repressor. RT-qPCR experiments measured *umuDAb* expression in wild type (WT) strains and *ddrR* mutant strains MSUcds2730 (* A. baylyi *) and JH1700 (* A. baumannii *), in the absence or presence of DNA damage (MMC, 2 μg ml^−1^). Mutation of *ddrR* resulted in the loss of the *umuDAb* repression that exists in WT cells in the absence of DNA damage. Additionally, expression of *umuDAb* after DNA damage was significantly induced in the ADP1 *ddrR* mutant MSUcds2730 (*P*<0.05 in a *t* -test). Asterisks indicate statistical significance in a Student’s *t* -test for *P*<0.05. The standard error of the mean from technical triplicates of biological triplicates is shown.

**Fig. 3.**
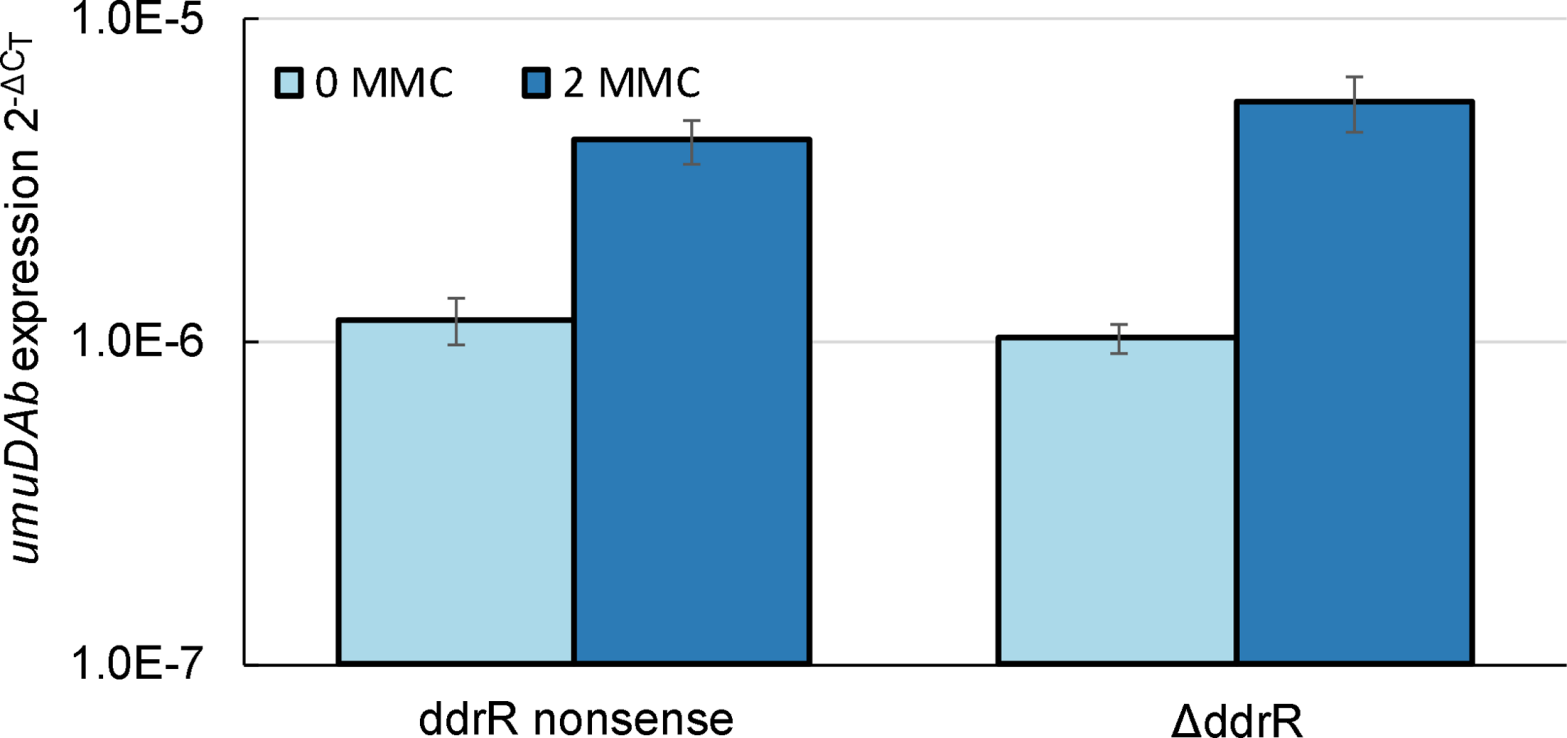
A DdrR protein probably exerts the regulatory actions of *ddrR.* RT-qPCR experiments measured the expression of *umuDAb* in two different *ddrR* mutants of ADP1 in the absence or presence of DNA damage (MMC, 2 μg ml^−1^). DR-Stop, a *ddrR* nonsense (stop codon) mutant, showed the same derepression of *umuDAb* in the absence of DNA damage as did the null *ddrR* mutant MSUcds2730. There was a significant induction in *umuDAb* expression (2^−Δ*C*T^) after DNA damage (*P*<0.01 in a one-tailed *t* -test) in each strain, but no significant difference between strains in their induction amount (*P*>0.05 in a two-tailed *t* -test comparing 2^−ΔΔ*C*T^), or between expression in either the presence or the absence of MMC (*P*>0.05 in a two-tailed *t* -test.) The standard error of the mean from technical triplicates of biological triplicates is shown.

**Fig. 4.**
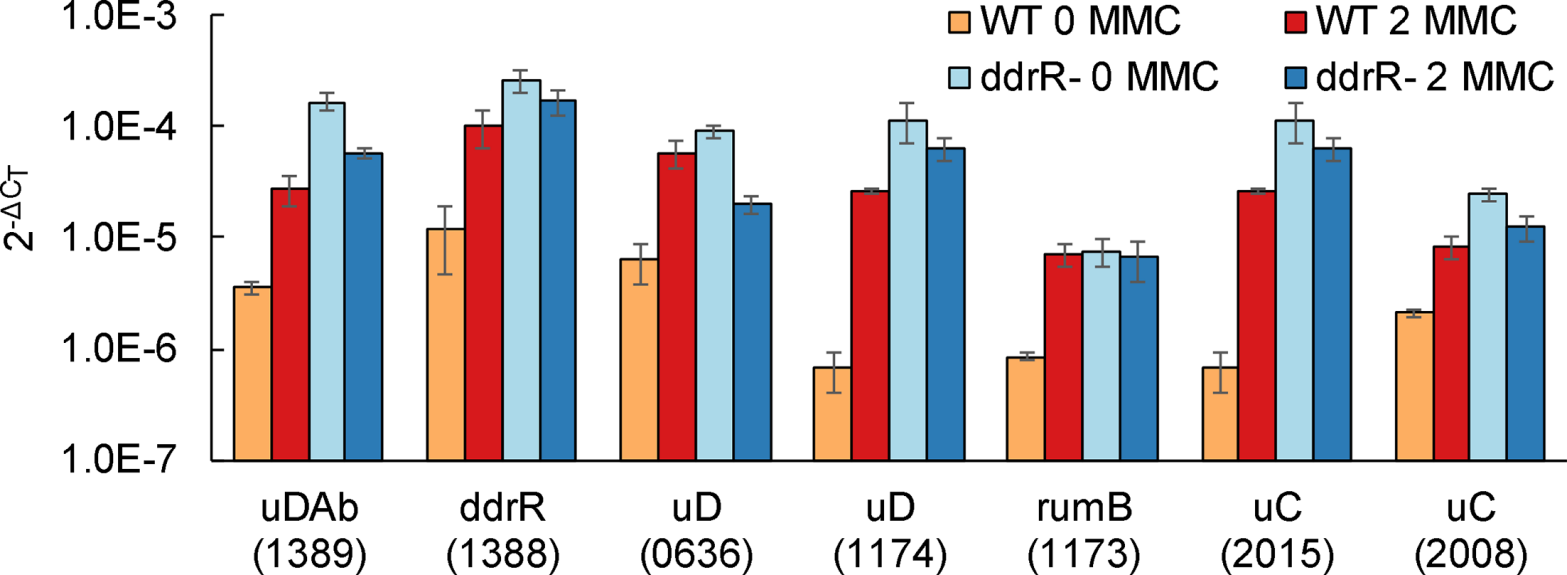
*ddrR* is required for repression of multiple error-prone polymerases in 17978. RT-qPCR experiments measured the expression of genes in 17978 WT and *ddrR* strain JH1700 in the absence or presence of DNA damage (MMC, 2 μg ml^−1^). Mutation of *ddrR* resulted in the derepression of all of these genes in the absence of DNA damage. Expression was measured in both un-induced and induced (MMC) conditions of WT or *ddrR* mutant cells. Genes are indicated by name or abbreviation (uDAb*, umuDAb*; uD*, umuD*; uC*, umuC*), and A1S gene locus number. Each gene was assayed in one RT-qPCR experiment (plate), with error bars indicating the standard error of the mean from technical triplicates of biological triplicates. For every gene, expression in WT cells was significantly (*P*<0.05 in a one-tailed *t* -test) increased after induction with MMC. Expression of every gene in the absence of DNA damage was significantly (*P*<0.05; in a one-tailed *t* -test) induced in *ddrR* mutant cells as compared to WT cells.

**Fig. 5.**
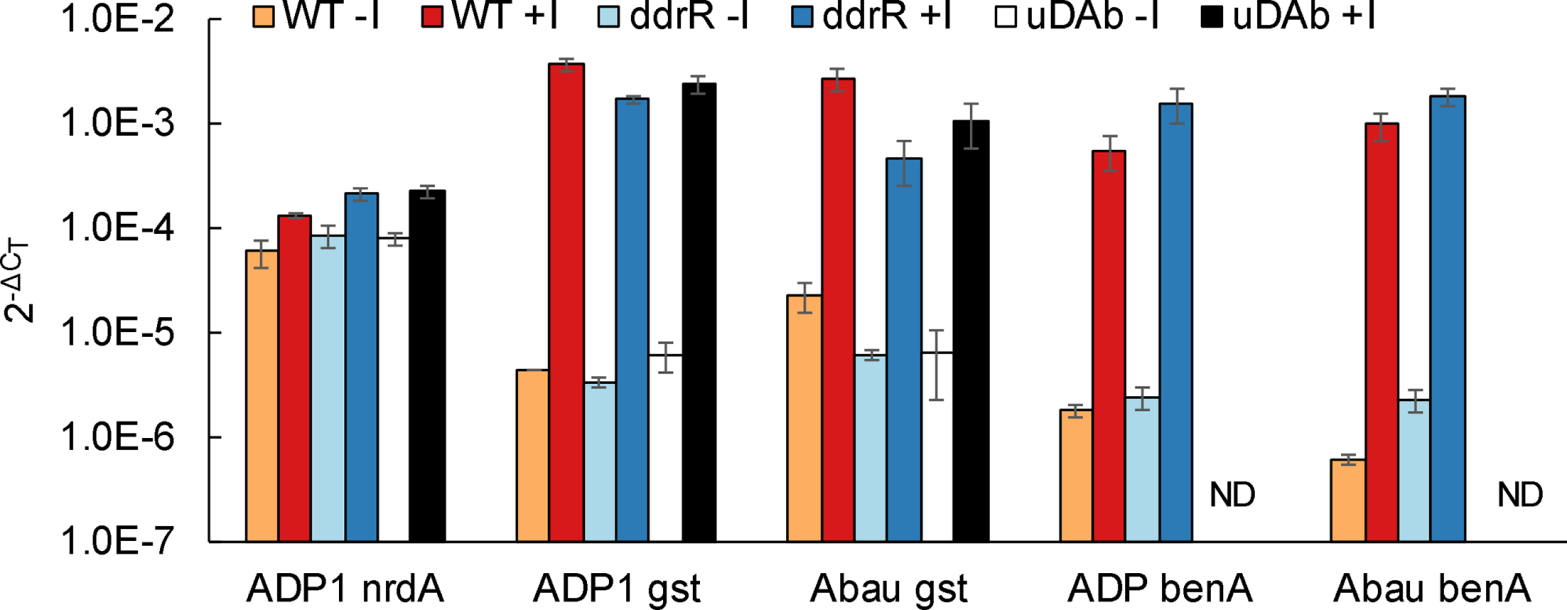
*ddrR* mutation does not affect the induction of genes that are not regulated by UmuDAb. RT-qPCR experiments measured the expression of genes (ACIAD0724 *nrdA*, ACIAD0445 *gst* or ACIAD1436 *benA* in ADP1; A1S_0408 *gst* or A1S_1215 *benA* in 17978) in un-induced (-I) and induced (‘+I’ for inclusion of inducing agent: MMC for *nrdA* and *gst;* or benzoate for *benA*) conditions for WT, *ddrR* and *umuDAb* strains of ADP1 and 17978. There was no significant difference in the induction level of any of these genes in the *ddrR* or *umuDAb* mutants (*P*>0.05; in a two-tailed *t* -test) after MMC or benzoate exposure (for *benA* gene expression only). nd *, benA* expression was not examined (not done) in *umuDAb* mutants of either 17978 or ADP1 by RT-qPCR, although previous experiments have shown that benzoate-mediated induction of *benA* is unaffected by the *umuDAb* mutation [18]. Each gene was assayed in one RT-qPCR experiment (plate), with error bars indicating the standard error of the mean from technical triplicates of biological triplicates.

GraphPad InStat software was used to conduct the ANOVA and *t* *-*test analyses as described in the text and legends for Figs 2–5.

## Results

*umuDAb*, as a *umuD* and *lexA* homologue [18], is a canonical SOS gene and hence its regulation by, and induction after, DNA damage was expected. However, the *ddrR* gene, which is highly induced after DNA damage [18], is of unknown function and does not encode a protein with homology to any other proteins. It is found only among members of the genus *Acinetobacter* [9], where every *umuDAb* allele and *ddrR* allele (which range in size from 231 to 252 bp) are co-located and oriented divergently from each other.

### *ddrR* and *umuDAb* promoters overlap with the UmuDAb binding site

We sought to determine how the one UmuDAb binding site suggested to exist in the *ddrR–umuDAb* intergenic region could accomplish the regulation of both *umuDAb* and *ddrR* expression. To map the relationship between the UmuDAb binding site and these genes’ putative promoters, we identified the transcriptional start sites of *ddrR* and *umuDAb* in *A. baylyi* strain ADP1 (ACIAD2730 and ACIAD2729) and *A. baumannii* ATCC 17978 (A1S_1388 and A1S_1389). We hypothesized that *ddrR* and *umuDAb* might have overlapping promoters that could allow one UmuDAb binding event to regulate both genes’ expression coordinately.

5′ RACE experiments for *ddrR* indicated a +1 site 41 bp upstream of *ddrR* in ADP1, and 180 and 182 bp upstream of *ddrR* in 17 978 (Fig. 1). 5′ RACE determined the +1 site for *umuDAb* transcription to be 30–31 bp upstream of *umuDAb* in ADP1 and 30 bp upstream of *umuDAb* in 17 978. These +1 sites for *ddrR* and *umuDAb* predict adjacent −35 promoter consensus elements for *umuDAb* and *ddrR* in both species.

These data, combined with previous data showing a loss of transcription when these bases have been mutated [14], suggest that UmuDAb simultaneously represses both genes by binding to DNA and blocking RNA polymerase access to both of the *umuDAb* and *ddrR* promoters. This coregulation of both genes suggested that the gene products, once expressed, might be used in the same pathway or process.

### *ddrR* encodes a protein that regulates gene expression after DNA damage

The coregulation of *ddrR* and *umuDAb* transcription by UmuDAb suggested that a *ddrR* mutant might, like a *umuDAb* mutant, also cause dysregulation or derepression of DNA damage-induced genes in *Acinetobacter *. This hypothesis was tested in RT-qPCR experiments performed on RNA harvested from ADP1 WT and MSUcds2730 (Δ*ddrR::tdk/kan*), and 17978 WT and JH1700 (*ddrR*:Tn*LK*) cells grown in inducing (2 μg MMC ml^−1^) and control conditions. Expression of *umuDAb* in these *ddrR* mutants and in the extended deletion mutant strain ACIAD2730 (data not shown; *P*<0.05) was significantly derepressed under control conditions, relative to the WT strains (*P*<0.05) (Fig. 2). In each species, *umuDAb* expression was significantly (*P*<0.05) higher in the *ddrR* mutant cells than in WT cells. In ADP1 but not 17978, *umuDAb* was further significantly induced (5.4-fold) in the Δ*ddrR* cells after MMC treatment (Fig. 2), even after the loss of repression in the absence of MMC treatment.

It is not known whether *ddrR*, uniquely found in the genus *Acinetobacter *, encodes a protein or whether it exerts its action as an RNA transcript. We mutated codon 4 of the ADP1 *ddrR* to form a stop codon and noted that the induction and expression of *umuDAb* (in both the absence and the presence of MMC) was the same in this ADP1 *ddrR* nonsense mutant strain (DR-Stop) as in the null *ddrR* mutant MSUcds2730 (Fig. 3). These results suggest that the changes in transcription seen in MSUcds2730 were due to the absence of a DdrR protein. Similar to *umuDAb* expression being equally induced in MSUcds2730 and DR-Stop, *ddrR* expression in DR-Stop also showed 2.11-fold induction (*P*<0.05 in a one-tailed *t* *-*test; data not shown) after MMC treatment.

We next tested whether *ddrR* regulation of *umuDAb* transcription was simply due to *umuDAb*’s proximity next to *ddrR*, or if DdrR also repressed itself and other genes in the UmuDAb regulon: the *umuC* error-prone polymerases and *umuD* polymerase managers *umuDC* A1S_0636–0637; *umuDrumB* A1S_1174–1173; *umuC* A1S_2008; and *umuC* A1S_2015 [13, 17]. RT-qPCR experiments in JH1700 showed significantly increased expression of *umuDAb* and its regulon in the absence of DNA damage (Fig. 4), as did *umuDAb* mutants of 17 978 on *umuDC* expression [17]. *ddrR* also repressed its own expression, as evidenced by the similar increase in its expression in JH1700 cells in the absence of DNA damage (Fig. 4).

Further RT-qPCR experiments in both species showed that *ddrR* did not, however, regulate the expression of all DNA damage-induced genes. Several DNA damage-induced genes that are not regulated by UmuDAb [17], such as the glutathione *S*-transferase gene *gst* (A1S_0408 and ACIAD0445) and the nucleotide reductase gene *nrdA* (ACIAD0724), were not regulated by *ddrR* either (Fig. 5). Additionally, benzoate-induced *benA* genes (ACIAD1436 and A1S_1215), whose expression is unaffected by the *umuDAb* mutation, were similarly unaffected by *ddrR* mutation in JH1700 (Fig. 5).

### The *ddrR* transcriptome shows coregulation of DNA-damage inducible genes with UmuDAb

To test whether *ddrR* also repressed (or otherwise regulated) additional genes, we conducted RNA-seq experiments on JH1700 cells that were either untreated or treated with MMC. Genes that were induced in this *ddrR* mutant more than two-fold after MMC treatment and had an FDR of less than 0.01 were considered to be differentially expressed (*n*=182). Roughly two-thirds of these (*n*=113) had been identified previously as MMC-inducible in WT cells, so their differential expression in JH1700 suggested that DdrR was not required for their induction. (None were derepressed in the absence of MMC treatment and further induced in the presence of MMC.) Most (95 %) of these DdrR-independent genes were located in the three cryptic prophages designated CP5, CP9 and CP14 [23] that contain ~90 % of the genes induced in the WT 17978 cells after MMC treatment [17].

**Fig. 6.**
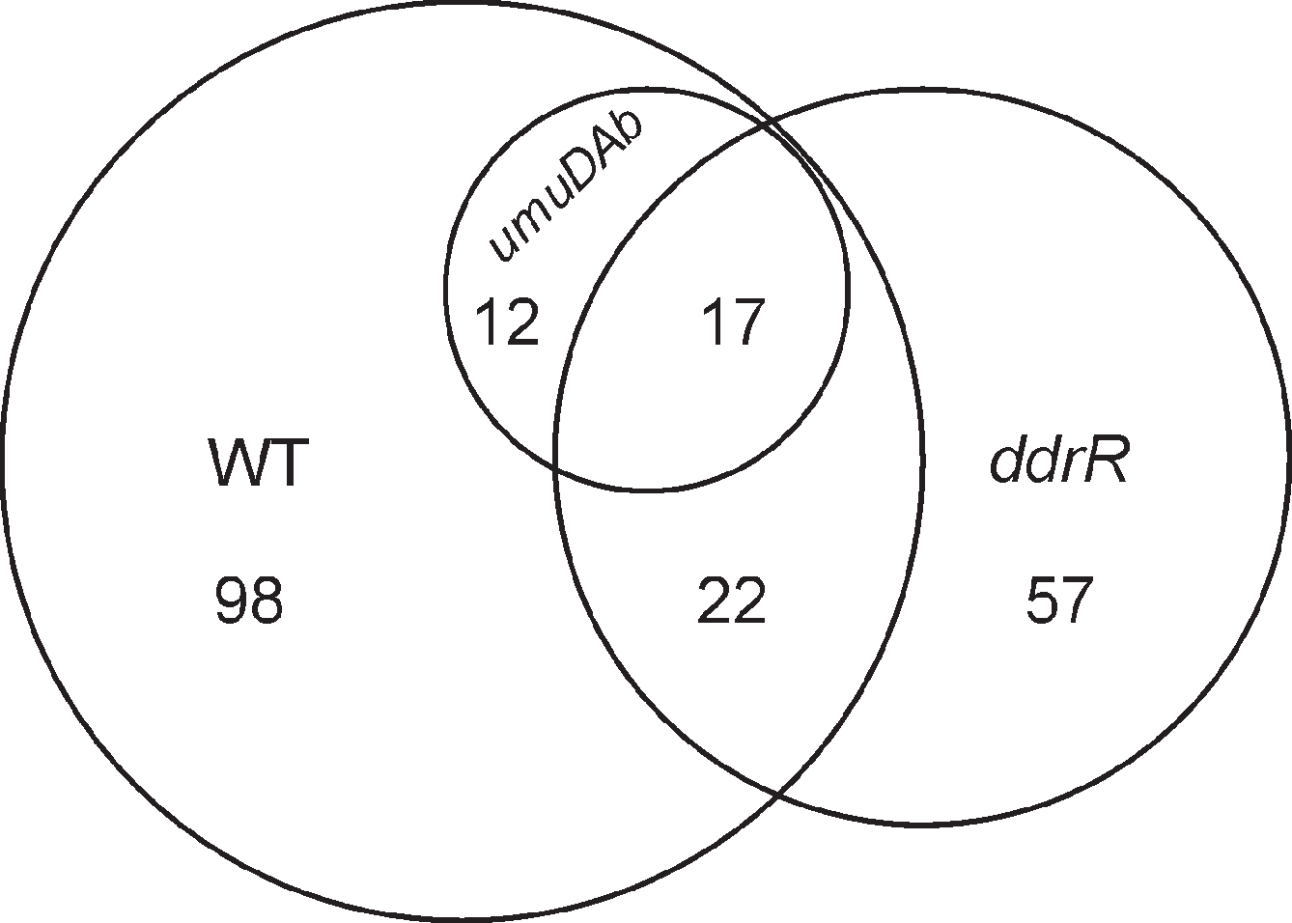
Proportion of the DNA damage-inducible genes that are regulated by *ddrR* and *umuDAb*. The relationship between 17978 genes induced after MMC in WT cells and *ddrR*- and *umuDAb*-dependent genes induced is shown in an area proportional Venn diagram constructed using the BxToolBox at bioinforx.com.

Approximately 25 % (*n*=39 genes) of the established MMC-induced regulon of WT 17978 cells [13, 17] was regulated by *ddrR* (i.e. the genes were not MMC-induced in JH1700 cells; Table 4). UmuDAb also regulated the expression of 17 of these 39 *ddrR*-regulated genes (Fig. 6), which were flagged for further study (Table 4). Within the *ddrR*- and *umuDAb*-regulon was a group of DNA damage-inducible genes that are repressed before DNA damage and are induced after repression is lifted. These include the *umuC* error-prone polymerases and *umuD* polymerase managers, as well as *umuDAb* and *ddrR*, all of which are repressed by UmuDAb before MMC treatment [13, 17]. These eight genes were the only DNA damage-inducible genes whose expression was derepressed in JH1700 cells in the absence of DNA damage. RT-qPCR experiments validated the derepression seen in the RNA-seq data of each of these genes (Fig. 4).

**Table 4.** DNA damage-inducible genes regulated by DdrR

Regulation	Gene locus*	Gene name/function	Location†	COG(s)‡
Repressed by DdrR and UmuDAb				
	A1S_1388	*ddrR*		
	A1S_1389	*umuDAb*		KT
	A1S_0636 A1S_0637	*umuD*	pAB3	KT
		*umuC*	pAB3	L
	A1S_1173 A1S_1174	*rumB*	CP5	L
		*umuD*	CP5	KT
	A1S_2008	*umuC*		L
	A1S_2015	*umuC*	CP9	L
Activated by DdrR and UmuDAb				
	A1S_0278	*trnT*; Trp tRNA		
	A1S_0421	*infA*; translation factor IF-1		J
	A1S_1144	repressor; S24 family peptidase	CP5	K, KT
	A1S_3622	hypothetical	CP5	N, T, M
	A1S_2014	SOS response-associated peptidase	CP9	S
	A1S_2037 A1S_3774 A1S_3775	*esvI*; transcriptional regulator/repressor	CP9	KT
		hypothetical	CP9	
		hypothetical	CP9	
	A1S_3704	holin	CP14	
Regulated by DdrR§				
	A1S_1147	site-specific DNA methylase-like	CP5	L
	A1S_3611 A1S_3612 A1S_3613 A1S_1148 A1S_1149 A1S_1151	hypothetical	CP5	
		HNH endonuclease	CP5	V
		hypothetical	CP5	
		hypothetical	CP5	S
		hypothetical	CP5	
		hypothetical	CP5	S
	A1S_3603	hypothetical	CP5	
	A1S_3604	hypothetical	CP5	
	A1S_3608	hypothetical	CP5	
	A1S_3615	hypothetical	CP5	L
	A1S_3621	hypothetical	CP5	GEPR
	A1S_2031	phage protein	CP9	S
	A1S_3755	holin	CP9	GEPR
	A1S_3772	hypothetical	CP9	
	A1S_3778	hypothetical	CP9	
	A1S_3779	hypothetical	CP9	
	A1S_3693	hypothetical	CP14	
	A1S_3695	hypothetical	CP14	
	A1S_3696	hypothetical	CP14	
	A1S_3699	hypothetical	CP14	
	A1S_3705	hypothetical	CP14	

*Gene loci appearing together in the same box indicate that these loci reside in the same operon.

†Where no location is given, a chromosomal location outside of a prophage is indicated.

‡COG, Cluster of Orthologous Groups; a method of functional annotation of genes based on orthology in complete microbial genomes [39].

§These genes were induced >2× after DNA damage, but their FDR values did not fall below 0.01 [or 0.05, with the exception of A1S_1149 (*q*=0.013) and A1S_3699, (*q*=0.016)], so they were not differentially expressed in the *ddrR* mutant and were thus considered to be regulated by DdrR.

Another group of nine DNA damage-inducible genes was co-regulated not through repression, but by being activated by both DdrR and UmuDAb. Expression of some of these genes did not increase in JH1700 cells after DNA damage. These included *infA* (translation initiation factor IF-1, A1S_0421) and three CP9 genes: *esvI* (putative phage repressor A1S_2037 [24]), a putative SOS response-associated peptidase (A1S_2014 [25]) and a hypothetical ORF (A1S_3774). The expression of the other five genes in this group increased more than two-fold after DNA damage, in both the *ddrR* and the *umuDAb* mutants (exception: A1S_0278, which was induced 1.8-fold in the *umuDAb* mutant), but were not considered differentially expressed. These showed, as a trend, lower expression after DNA damage than in WT cells. These included a putative CP5 phage repressor (A1S_1144), the tRNA gene *trnT* (A1S_0278) and three hypothetical ORFs located in the cryptic prophages (Table 4).

### DdrR regulates some DNA-damage inducible genes without UmuDAb cooperation

Our analysis also identified 22 DNA damage-inducible genes whose induction was regulated by DdrR but not UmuAb (Table 4). These were located in the three cryptic prophages CP5, CP9 and CP14 and encoded mostly hypothetical phage proteins (Table 4). They were dependent upon RecA for their induction, like 99 % of the DNA damage-inducible genes in 17 978 [17]. These genes were not differentially expressed after DNA damage in JH1700 cells, although they were all induced more than two-fold (median 6.5-fold induced) and displayed lower expression in the absence of MMC treatment than in WT cells (*P*<0.05 in a *t* -test).

As a test of whether the lower expression in JH1700 was merely a characteristic of this strain, we evaluated the expression of 20 randomly chosen genes that were not DNA damage-inducible (Table S2). There was no significant difference (*P*>0.2 in a non-parametric repeated-measures ANOVA using a Friedman test) between expression of these genes in the WT and JH1700 strains in either the control or the MMC-treated condition.

### DdrR also regulates genes that are not DNA damage-induced in WT cells

A fourth set of *ddrR*-regulated genes was differentially expressed and induced in JH1700 but had not been induced in WT cells (*n*=69). Further analysis comparing these *ddrR* RNA-seq reads to FPKM reads from a previous RNA-seq study [17] and also to a microarray study [13] indicated that 12 of these genes were induced by MMC in WT cells (five genes were induced in both studies). This reduced the number of *ddrR*-dependent, inducible genes to 57 (Table 5). These were largely chromosomally encoded, except for two genes on pAB3, and outside of cryptic prophages (except for three CP14 genes). This regulon contained multiple genes related to DNA replication, recombination and repair: a helicase, *recX*, *ruvA, ruvB*, *parE* and others (see Table 5 for gene identities). Multiple genes were identified as transcriptional regulators: a phage repressor A1S_1582 in CP14, as well as four AraC family, an AsnC family and an AcrR/TetR family regulators. General resistance–nodulation–cell division (RND) efflux (A1S_0008, 3146), RND transporters (*adeFGH*; A1S_2304–2306) and other transport functions (A1S_0023, 0030, 2068–2070, 2977) were also commonly represented.

**Table 5.** Genes induced after DNA damage in *ddrR* mutant but not induced in WT

Gene locus*	Name/function	COG
A1S_0005†	put.‡ cytochrome b precursor	C
A1S_0008	put. RND type efflux pump	P
A1S_0023	put. malic acid transport protein	P
A1S_0030	alkanesulfonate transport protein	P
A1S_0094	*lrp* regulon transcriptional regulator (AsnC family)	K
A1S_0170	put. outer membrane copper receptor (OprC family)	P
A1S_0276† A1S_0277†	*trnY*	
	*trnG*	
A1S_0310	excinuclease ABC subunit C (*esvL*; ethanol-stimulated virulence protein [24])	L
A1S_0422†	put. transcriptional regulator (AraC family)	K
A1S_0564	put. translation initiation inhibitor (*yjgF* family)	J
A1S_0663	put. DNA helicase on plasmid pAB3	L
A1S_0666	TrbL/VirB6 plasmid conjugal transfer protein on plasmid pAB3	U
A1S_1124	transcriptional regulator (AraC family)	K
A1S_1377†	transcriptional regulator (AcrR family)	K
A1S_1383 A1S_3661	surface Ag	
	hypothetical	
A1S_1582†	put. bacteriophage repressor C2 of prophage CP14	K, KT
A1S_1614†	hypothetical	S
A1S_1746	put. transcriptional regulator	K
A1S_1761	acetyltransferase	KR
A1S_1762†	hypothetical	K, E
A1S_1963	*recX* regulatory protein	R
A1S_2068	put. benzoate membrane transport	Q
A1S_2069 A1S_2070†	put. Mg^2+^ transporter transmembrane	S
	*mgtA*; P-type ATPase Mg^2+^ ATPase transporter	P
A1S_2148 A1S_2149† A1S_2150	put. acetyl-CoA synthetase/AMP-(fatty) acid ligase	I
	put. acyl CoA dehydrogenase oxidoreductase	I
	oxidoreductase short-chain dehydrogenase/reductase family	IQR
A1S_2304 A1S_2305 A1S_2306	*adeF* RND family drug transporter	M
	*adeG* cation/multidrug efflux pump	V
	*adeH* put. RND family drug transporter	MU
A1S_2586 A1S_2587 A1S_2588†	dGTP triphosphohydrolase	F
	*ruvA* Holliday junction helicase subunit A	L
	*ruvB* Holliday junction helicase subunit B	L
A1S_2963†	*purK*; phosphoribosylaminoimidazole carboxylase ATPase subunit	F
A1S_2970	put. glutathione-like synthetase	E
A1S_2977	cation diffusion facilitator family transporter	P
A1S_3139†	put. signal peptide	
A1S_3146†	*mdfA*; Multidrug efflux transport protein	GEPR
A1S_3326	put. membrane protein	S
A1S_3359†	*parE*; topoisomerase IV subunit B	L
A1S_3361	hypothetical	R, R
A1S_3385†	put. membrane protein	
A1S_3428 A1S_3429	put. glucose dehydrogenase precursor	G
	hypothetical	S
A1S_3472	plasmid replication (pAB2)	L
A1S_3485	hypothetical	S
A1S_3563	hypothetical; between *nrdA* and *nrdB*	
A1S_3574†	peptide between 30S ribosomal proteins *rpsG* (S7) and *rpsL* (S12)	
A1S_3662	general stress protein	R
A1S_3690†	hypothetical (CP14)	
A1S_3692†	hypothetical (CP14)	
A1S_3709	hypothetical	
A1S_3865	hypothetical	
A1S_3877†	hypothetical peptide	

*Gene loci appearing together in the same box indicate that these loci reside in the same operon.

†These genes were also induced in the *umuDAb* mutant but not in WT 17978 cells [17].

‡“put.” = Putative function.

Finally, 28 genes were repressed in JH1700, 14 of which were previously observed to be repressed in WT cells [17] (Table 6). In all but two genes out of these 28, the expression pattern (of being repressed or not repressed) was the same in the *umuDAb* mutant as in WT cells. None of these genes were located in the cryptic prophages.

**Table 6.** *
A. baumannii* genes repressed in the *ddrR* mutant

Gene locus	Name/function	Log_2_-fold change in *ddrR* mutant	Repressed in wild type*?
A1S_0292	put. outer membrane protein W	−1.69	yes
A1S_0391	50S ribosomal protein L31 type B	−2.70	
A1S_0548	put. transcriptional regulator (TetR family)	−1.50	yes
A1S_0549	hypothetical	−1.42	yes
A1S_0891	hemerythrin-like metal-binding protein	−1.71	yes
A1S_1216 A1S_1217 A1S_1218	LysR regulator	−1.49	
	heavy metal translocating P-type ATPase	−2.60	
	*hmrR*, copper-responsive HTH regulator (MerR family)	−1.99	
A1S_1319	hypothetical	−1.59	*
A1S_1467	put. glutamate symport transmembrane protein	−1.54	
A1S_1476	hypothetical	−1.30	
A1S_1734	hypothetical	−1.31	
A1S_1811	ankyrin-related protein	−1.26	
A1S_1924 A1S_1926	*cydA*, cytochrome d terminal oxidase	−2.00	yes
	put. membrane protein	−2.12	yes
A1S_1927	put. acetyltransferase	−1.24	*
A1S_2098	put. alcohol dehydrogenase	−1.77	
A1S_2102	aldehyde dehydrogenase 1	−1.31	
A1S_2210	hypothetical	−1.69	
A1S_2317	putative lipoprotein precursor (RlpA-like)	−2.03	yes
A1S_2936	copper resistance protein A precursor	−2.11	
A1S_3402 A1S_3403 A1S_3404	*rocF* hydrolase	−1.69	yes
	*hutI* imidazolonepropionase	−1.65	yes
	proline transport protein (APC family)	−1.39	yes
A1S_3627	hypothetical	−2.15	yes
A1S_3748	hypothetical	−1.24	yes
A1S_3794	hypothetical	−1.68	yes
A1S_3858	hypothetical	−1.73	yes

*Response for UmuDAb regulatory status is the same as for WT cells, except for where an asterisk symbol appears, denoting that the gene is also repressed in *umuDAb* mutant cells.

## Discussion

### Defining the source of *ddrR*–*umuDAb* transcriptional coregulation

Based on the overlap of the ADP1 inverted repeats (IRs) [18] with the *ddrR* and *umuDAb* −35 promoter consensus elements, and the similar overlap of these −35 elements with the 17 978 UmuDAb binding site [13], we propose that UmuDAb binding to DNA concurrently represses the transcription of both of these genes by blocking RNA polymerase access. In a previous study, *A. baylyi* strain JH100 showed no induction of *ddrR* and *umuDAb* expression under DNA damaging conditions [14]. JH100 possesses mutations in what this study defined as the *ddrR* −35 consensus element (changing it from TTGACG to GAGACG), and the *umuDAb* −35 consensus element (from TTGAAT to CAACGT). These mutations in the −35 elements are the most likely explanation for this loss of gene expression in this strain. The newly identified *ddrR* −35 element includes bases of the IRs identified in our previous study [18], which are identical in both species (Fig. 1), although it does not overlap the UmuDAb binding site proposed for 17978 cells [13]. These results also suggest that the required UmuDAb binding region may be larger than previously suggested [13]. It is interesting that the σ^70^ −35 promoter consensus elements are not located in the two separate IRs that compose the UmuDAb binding site, which contains TTGAA(A/T) inverted repeats. Rather, both of the −35 elements overlap the same IR, which is farthest from *umuDAb*. This arrangement allows the tightly coordinated expression of DdrR and UmuDAb. Our observations are consistent with the fact that other repressors (e.g. LexA) do not always bind to the same area within a promoter. Sometimes the repressor binding site overlaps with the −35 or −10 element, while other promoters contain the binding site between these two elements, downstream of the promoter elements, or even in the ORF itself [26]. The UmuDAb binding site was similarly placed upstream of *umuDAb* in both species but a variable distance upstream of *ddrR* as there is a longer intergenic region present in 17 978 relative to ADP1.

### DdrR coregulates multiple genes with UmuDAb

Repression of six error-prone *umuDC* homologues, *umuDAb* and *ddrR* required both DdrR and UmuDAb. The *ddrR* mutation caused *umuDAb* overexpression but also yielded more expression of *rumB*, *umuD*, *umuC*, *umuDAb* and *ddrR*, which are repressed by UmuDAb. These observations suggest that DdrR might act as a corepressor to aid in UmuDAb repressive DNA binding. The UmuDAb N-terminal domain, which may possess a helix-turn-helix (HTH) structure like the DNA damage response repressor LexA, is required to repress DNA damage-induced genes [14]. In this model, the lack of DdrR might prevent UmuDAb repression from these genes’ promoters in a manner consistent with our observations. This action of *ddrR* suggests that DdrR is a corepressor, with UmuDAb, of a specific set of error-prone polymerases in *Acinetobacter *. The DdrR protein did not repress all DNA damage-inducible genes, but only corepressed the UmuDAb-repressed regulon. These results represent the first example of a LexA-like repressor using a corepressor to control host chromosomal genes (namely, A1S_2008, 1388 and 1389), although LexA often regulates horizontally acquired genetic elements such as plasmid or prophages via corepressors that may respond to nutritional or environmental factors [27–30].

The strength of the connection between DdrR and UmuDAb regulatory action was seen not just in *ddrR* corepressing UmuDAb-repressed genes, but also in *ddrR* coregulating genes that require UmuDAb for their increased expression after DNA damage. UmuDAb has a role in repressing transcription of specific genes before DNA damage [12, 14, 17], where UmuDAb self-cleavage at a conserved C-terminal site relieves this repression and induces gene expression [6]. However, some evidence suggests additional possible roles in the DNA damage response system of *Acinetobacter *, specifically in allowing or causing induction of genes after DNA damage [13]. In this study, besides the DdrR–UmuDAb corepressed genes, nine genes were coregulated by DdrR and UmuDAb, showing no significant increase in target gene expression after DNA damage in both the *ddrR* and *umuDAb* mutants. This suggests an additional coregulatory process involving both genes’ products where DdrR may act together with UmuDAb in this role as well as its actions as a repressor.

In the coregulated class of nine DNA damage-inducible proteins, it is striking that three are either phage repressors [A1S_1144 and A1S_2017 (*esvI*), an ethanol-stimulated virulence factor identified in a *Caenorhabditis elegans* screen of 17 978 [24]] or putatively associated with DNA damage sensing (A1S_2014). A1S_2014 was identified through comparative genetics as a member of a new SOS response-associated peptidase (SRAP) family [25], and is transcribed upstream of the *umuC* homologue A1S_2015, but was regulated differently from it in this study. These similarities suggest that besides UmuDAb, DdrR might work with additional repressors or regulatory proteins in a broader DNA stress response. Additionally, three of these nine genes appear to be, based on analyses of conserved gene pairs with OperonDB [31], encoded as part of the same CP9 operon: *esvI* and two hypothetical proteins A1S_3774 and 3775. DdrR and UmuDAb also coregulate the gene encoding initiation factor IF-1 (A1S_0421), which is responsible for translational initiation [32]. Mutation in either *ddrR* or *umuDAb* might result in an inability to induce the amounts of IF-1 needed to produce sufficient DNA damage response proteins to respond to DNA damaging conditions.

Compared to the *ddrR*- and *umuDAb*-coregulated genes, it is possible that the functions of the genes regulated only by *umuDAb* require less tight repression and earlier expression than the mutagenic, error-prone polymerases corepressed by *ddrR*, which are typically expressed late in the SOS response [33]. Alternatively, it may be that *ddrR* responds to an additional nutritional signal that is not related to UmuDAb-regulated genes that carry out different functions. This would be consistent with corepressor actions seen previously [27, 28, 34].

### Regulation by DdrR can occur without UmuDAb involvement

This study showed that some (*n*=22) DdrR-regulated genes are not codependent on UmuDAb. In total, 99 % of the 17 978 DNA damage-inducible transcriptome [12, 17], and all but one of the 28 UmuDAb-regulated genes, are dependent upon RecA for their regulation (the notable exceptions being *recA* itself, and the hypothetical phage gene A1S_2020).

Finally, one-third (*n*=20) of the 57 genes whose expression was differentially induced in JH1700 cells but not in WT cells were located in seven potential operons or gene clusters of two or more adjacently transcribed genes. These operons are predicted by the OperonDB database [31] and/or MicrobesOnline predictions [35], which further supports the validity of this classification of *ddrR*-dependent genes. These gene clusters included a triphosphohydrolase and Holliday junction helicase genes *ruvAB* A1S_2586–2588, Mg^2+^ transport genes A1S_2069–2070, fatty acid ligase and oxidoreductases A1S_2148–50, RND efflux transporters A1S_2304–2306, and multiple genes of unknown function. The A1S_2304–2306 operon is significant because these are known virulence genes in *A. baumannii* that when overexpressed are associated with biofilm formation and drug resistance [36] and induced by non-DNA damage stresses such as NaCl [37]. Using FPKM comparisons to an earlier study [17], we observed that 20 of these 57 genes had also been induced in a *umuDAb* mutant but not in the WT cells. These did not constitute particular clusters of genes, however, with typically one, or none, of three genes in a cluster showing regulation by UmuDAb. This induction was *recA*-dependent in nearly all of these 57 genes except A1S_1383 and A1S_3661, which were identified as co-located in an operon, and the hypothetical A1S_3877. This RecA-dependence allows for the possibility that DdrR may be working through a RecA-sensitive DNA damage-sensing component, or is itself RecA-sensitive.

In these experiments, we examined the role of the *ddrR* gene in two different *Acinetobacter* species. Both the model soil microbe *A. baylyi* ADP1 and the opportunistic pathogen *A. baumannii* strain 17978 show corepression of the regulatory pair *ddrR–umuDAb* in the absence of DNA damage. However, the *A. baylyi ddrR* mutant strains, which expressed *umuDAb* in the uninduced condition similar to that of WT cells after DNA damage (Fig. 3), still further induced expression of these genes from that higher expression level. 17978 cells showed no additional induction in any UmuDAb-DdrR corepressed gene. As ADP1 does not possess any *umuDC* operons or unassociated *umuC* gene targets of DdrR–UmuDAb repression, we could not directly test whether this observation extended to other genes in ADP1, as we could for 17 978. One speculation is that a DdrR–UmuDAb–promoter physical interaction is stronger in *A. baumannii *, perhaps due to selection for tighter control of its several *umuDC* error-prone polymerases. DdrR and UmuDAb are 60 and 79% identical between the two species, respectively, allowing for this possibility.

The coordinate DdrR–UmuDAb repression of 17 target genes in *A. baumannii *, and of *ddrR–umuDAb* in both species, is facilitated by the joint auto-regulation of DdrR and UmuDAb. In our RNA-seq analyses, we identified multiple regulons of DdrR-regulated genes, including a regulon corepressed in conjunction with UmuDAb and a regulon coregulated with UmuDAb, which lacked induction when either protein was absent. In addition, DdrR regulated a group of 22 genes that do not depend on UmuDAb for their increased expression after DNA damage, as well as a group of 57 genes that were only differentially regulated (and induced) when *ddrR* was mutated. The unusual and atypical mechanisms and genes that this pathogen uses to control and cause its mutagenic responses to DNA damage highlight the importance of deciphering the response system used by the genus *Acinetobacter *.

## Supplementary Data

Supplementary material 1
