# Supplementary material for: A corepressor participates in LexA-independent regulation of error-prone polymerases in Acinetobacter
Source: Microbiology (Reading). 2019 Nov 5;166(2):212–26. doi: 10.1099/mic.0.000866 (PMC7273328; doi:10.1099/mic.0.000866)
Supplement: Supplementary material 1 [file mic-166-212-s001.pdf]

Supplemental Table 1. RT-qPCR primers

Gene locus*	Forward primer	Reverse primer
A1S_0408 (*gst*)	TGGGAAAGTGCCTGTATTGGT	TTCGGATCATCTAAAGCAGGTGC
A1S_0636 (*umuD*)	AAGTTGTTGCTCGGTCTCGG	TGCCGGTGATGGAAATCCTG
A1S_0637 (*umuC*)	TCTGAATCAATGCGCTCAAGACA	TCAAGTGCGGATAGGTTTTTGCT
A1S_1173 (*rumB*)	CGTTTCTGAAGTTTGGGGCG	CGTAGATCGTGCGAGCCATA
A1S_1174 (*umuD*)	CTCTCGACATGAACGAGCAC	CCGTGTTTGGCATCAAGACT
A1S_1388 (*ddrR*)	GGAAAGTGAAGCAGCCGAAAG	TGGGTAAGGGGATGTAAGCCT
A1S_1389 (*umuDAb*)	TACCACATTCCTTTGGCGAC	TCCGGCATCTAACATGGACA
A1S_2008 (*umuC*)	TGGAAGACCTCATGCAACAGACA	GCGTGCCATGACCACAGAAA
A1S_2014	CTGTTCACGAAAAGCCCAGC	TTTGCGATAAATTCCCCACCAT
A1S_2015 (*umuC*)	TTTGCACACTCTAGCCCGTT	TCAAGCGCAGTCAAAACCAC
A1S_2037 (*esvI*)	TGGTTGAAGAGTGGCTCCCT	GGACCACAACCATATCGCCA
ACIAD 0724 (*nrdA*)	ATGACCGTCGTCGTACTCAC	GCTGTGCAAATTCTTCGCCA
ACIAD 0445 (*gst*)	ACCTGTACTCACTGATGGCG	ACAGACCTCGTTTCGGATCA

*Gene prefix A1S = *A. baumannii*; prefix ACIAD = *A. baylyi*

Supplemental Table 2. Expression in RNA-Seq experiments of randomly chosen genes not part of the 17978 DNA damage response regulon, in WT *vs. ddrR* mutants

**A1S gene locus chosen randomly***	**Expression in untreated WT cells**	**Expression in untreated JH1700 cells**	**Expression in MMC-treated WT cells**	**Expression in MMC-treated JH1700 cells**
729	152.54	77.31	150.68	75.74
3349	631.83	692.09	481.64	731.41
2962	410.73	468.01	403.51	670.14
2219	70.46	26.69	72.73	17.32
3525	44.72	138.26	56.25	146.30
1033	107.99	38.39	117.27	58.84
1619	499.49	336.28	386.38	235.28
2943	106.28	19.62	139.60	30.89
743	107.05	19.36	103.94	28.48
268	3150.59	3932.55	1801.98	2567.42
694	87.64	31.56	95.67	33.41
635	67.94	42.03	123.00	57.49
425	71.20	40.04	88.67	46.42
1658	121.47	210.52	86.05	131.99
827	983.49	952.17	432.43	1049.13
2915	457.76	598.83	243.62	527.45
2736	1136.15	1725.66	440.62	1588.37
3505	155.72	94.91	157.47	99.85
300	60.92	44.47	96.72	57.46
1649	78.27	73.95	74.92	46.17

*Twenty random numbers between 1 and 3877 inclusive (corresponding to the A1S gene locus identifiers of the 17978 genome) were chosen by the Google random number generator.
